# Genetic diversity and drug susceptibility patterns of the *Mycobacterium tuberculosis* complex in Yunnan, China

**DOI:** 10.1042/BSR20181746

**Published:** 2019-05-31

**Authors:** Ruolan Bai, Shuijing Chi, Xiaofei Li, Xiting Dai, Zhenhua Ji, Miaomiao Jian, Yunfeng Bi, Feng Wang, Zhe Ding, Lisha Luo, Taigui chen, Aihua Liu, Fukai Bao

**Affiliations:** 1 Department of Biochemistry and Molecular Biology, Kunming Medical University, Kunming 650500, China; 2 Department of Microbiology and Immunology, Kunming Medical University, Kunming 650500, China; 3 Department of Laboratory Medicine, The Third People’s Hospital of Kunming, Kunming 650020, China; 4 The Institute for Tropical Medicine, Kunming Medical University, Kunming 650500, China; 5 Yunnan Demonstration Base of International Science and Technology Cooperation for Tropical Diseases, Kunming 650500, China; 6 Yunnan Province Integrative Innovation Center for Public Health, Diseases Prevention and Control, Kunming Medical University, Kunming 650500, China

**Keywords:** drug susceptibility, genetic diversity, Mycobacterium tuberculosis, spoligotyping

## Abstract

Tuberculosis (TB) is an infectious disease caused by *Mycobacterium tuberculosis* (Mtb) which has been threatening global public health for many years. High genetic diversity is dominant feature of Mtb. Increasing cases of multidrug-resistant (MDR) tuberculosis (MDR-TB) is a serious public health problem to TB control in China. Spontaneous mutations in the Mtb genome can alter proteins which are the target of drugs, making the bacteria drug resistant. The purpose of the present study was to analyze the genotype of Mtb isolates from some areas in Yunnan, China and explore the association between genotypes and MDR-TB. Using spoligotyping, we identified Beijing genotypes, six non-Beijing genotypes and a number of orphan genotypes from 270 Mtb isolates from patients in Yunnan Province during 2014–2016. Of 270 Mtb isolates, 102 clinical Mtb strains were identified as drug-resistant (DR) by drug susceptibility testing (DST), among them, 52 MDR strains. Beijing genotypes occupied the highest MDR proportion (78.85%) followed by the orphan genotypes (15.38%). The characteristics of MDR strains showed high genetic diversity. The results will help to efficiently improve diagnosis and treatment and provide valuable information for Mtb molecular epidemiology.

## Introduction

Under persistent threat of drug-resistant (DR) tuberculosis (DR-TB), 480000 cases of multidrug-resistant (MDR) tuberculosis (MDR-TB) emerged in 2015, and the countries with the largest numbers of MDR-tuberculosis (TB) cases were China, India, and the Russian federation (47% of the global total) with nearly a quarter of MDR-TB worldwide burden in China [1]. Findings from a recent national survey in China demonstrated that the prevalence of smear-positive tuberculosis is 19% [2]. According to a recent survey in China, it was demonstrated that the percentage of MDR-TB cases is 16.7%, and extensively DR-TB cases is 3.4% [3]. Some studies have suggested that relatively serious TB prevalence may occur in Western and Southwestern China [4]. Yunnan Province, as one of the areas with the most serious TB prevalence, is located on the Southwestern Chinese border [5]. Yunnan stands at China’s border with Southeast Asia and South Asia (the point at which India is located) and is a famous tourist resort that attracts many tourists from all over the world. Extensive MDR-MTB transmission would be more likely to occur among tourists if epidemic genotypes with MDR transmission dynamic observed in the study spread uncontrollably.

It has been found that the density of Beijing strains is higher than half of the *Mycobacterium tuberculosis* (Mtb) isolated from China [12,18]. The researchers hypothesized that Beijing strains have higher mutation rates, hypervirulence [6], treatment failures, and drug resistance [7]. However, such studies have not been published in Yunnan, and we hypothesized that the genotypes and anti-TB drug-resistance from patients with MDR-TB would be related.

## Materials and methods

### Study population

From July 2014 to December 2016, Mtb isolates were cultured from adults years of age with TB from The Third People’s Hospital in Yunnan Province, China. The participants were composed of 164 males and 106 females, with ages ranging from 15 to 82 years. The clinical specimens were mainly from sputum, but some form hydrothorax, ascites. All patients were diagnosed as new TB cases or retreatment TB cases. A total of 270 *Mtb* isolates from individual patients were subject to drug susceptibility testing (DST) using the BD BACTEC™ MGIT™ 960 automated rapid MTB culture system, which has been internationally accepted for this type of testing [8].

### Species identification and DNA extraction

Species identification was done in accordance with World Health Organization recommendations. Drug susceptibility was tested using the absolute concentration method. Tested drugs included rifampin (RIF; concentration 1.0 μg/ml), isoniazid (INH; 0.1 μg/ml), ethambutol (EMB; 5.0 μg/ml), streptomycin (SM; 1.0 μg/ml), and pyrazinamide (PZA; 100 μg/ml). Deoxyribonucleic acid (DNA) extraction of Mtb isolates was performed using the phenol/chloroform/isomyl alcohol method, purified DNA samples were followed by spoligotyping analysis [9].

Genomic DNA was extracted from freshly cultured bacteria manually. In detail, the bacterial cells were transferred into a microcentrifuge tube containing 0.01 M Tris/EDTA (TE) buffer (pH 8.0), followed by centrifuging at 13000 rpm for 2 min, the supernatant was discarded, and the pellet was resuspended in 500 ml TE buffer, followed by heating in 95°C water bath for 1 h and centrifuged at 13000 rpm for 5 min. The DNA in the supernatant was used for PCR amplification [10].

### Spoligotyping typing

Spoligotyping which relies on the highly polymorphic DR (direct repeat) locus in the genome of Mtb, is a method for genotyping of Mtb. First, DNA amplification is done by PCR system. Subsequently, the amplified PCR products were hybridized with Biodyne C membrane (Ocimum Biosolutions, Hyderabad, India) which have covalently linked 43 spacer oligonucleotides. Finally, the hybridized fragments were identified using enhanced chemiluminescence system (GE Healthcare, UK Ltd., Buckinghamshire, U.K.). The research was performed according to the method described by Kamerbeek et al. [9].

### Statistical analysis

Spoligotyping data were analyzed using the SITVIT database (http://www.pasteur-guadeloupe.fr:8081/SITVIT_ONLINE/) by BioNumerics 5.0 software to determine the spoligotype international type (SIT) [11]. The patterns were compared by the unweighted pair-group clustering method of averages [12]. Using the χ^2^ test or the Fisher’s exact test for sample size values of ≤5 for statistical analysis by SPSS software, differences were considered significant when the *P*-value was <0.05.

## Results

After using the spoligotyping database, the analysis of 270 clinical isolates revealed a very high genetic diversity of 68 different spoligotypes. Among these, 199 strains (73.70%) belonged to Beijing genotype, while the other 71 strains (26.30%) belonged to the non-Beijing genotype. Six genotypes were identified among the non-Beijing genotype isolates: (1) H3 (0.37%), (2) U (0.74%), (3) MANU2 (3.70%), (4) T1 (6.30%), (5) T2 (0.74%), (6) T3 (0.37%), and orphan genotypes which accounted for 38 (14.07%) of isolates, which were listed (Table 1).

**Table 1:** Frequencies of genotypes by family in *Mtb* isolates

Family		Number (%) of isolates	Number with susceptible isolates (%)	Number with drug-resistance isolates (%)	Number with susceptible isolates/number with MDR isolates	Number with drug-resistance isolates/number with MDR isolates
Lineage	Sublineage					
Non-Beijing		71 (26.30%)	45 (16.67%)	26 (9.63%)	45/11	26/11
H3		1 (0.37%)	1 (0.37%)	0	1/0	0/0
U		2 (0.74%)	2 (0.74%)	0	2/0	0/0
MANU2		10 (3.70%)	6 (2.22%)	4 (1.48%)	6/1	4/0
T		20 (7.41%)	15 (5.56%)	5 (1.85%)	15/2	5/2
	T1	17 (6.30%)	13 (4.81%)	4 (1.48%)	13/2	4/2
	T2	2 (0.74%)	2 (0.74%)	0	2/0	0/0
	T3	1 (0.37%)	0	1 (0.37%)	0/0	0/0
Orphan		38 (14.07%)	21 (7.78%)	17 (6.30%)	21/8	17/8
Beijing		199 (73.70%)	123 (45.56%)	76 (28.15%)	123/41	76/41
Total		270	168	102	168/52	102/52

Three results were obtained regarding the present study. First, among 270 patients with TB, the overall proportions of drug resistance to SM, INH, RIF, EMB, and PZA were 14.4% (39/270), 27.8% (75/270), 20.7% (56/270), 12.6% (56/270), and 18.5% (50/270), respectively, in Yunnan. Among Beijing genotypes, which constituted the largest proportion of genotype in the isolates, the proportions of drug resistance to SM, INH, RIF, EMB, and PZA were 15% (30/199), 28.6% (57/199), 22.1% (44/199), 13.6% (27/199) and 20.1% (40/199), respectively (Table 2). Second, out of these isolates, 52 (19.3%) isolates were classified as MDR [1] (MDR-TB is TB that is resistant to both rifampicin and INH, the two most powerful anti-TB drugs), and the proportion of Beijing genotype isolates was 78.8% (41/52) out of total MDR. Remarkably, for MDR-TB, smear-negative cases were twice as many as smear-positive cases. However, there were no significant differences in drug susceptibility profiles between Beijing and non-Beijing families when comparing DST results between these two groups of families. Third, from the perspective of DR distribution in each of the genotype strains, the highest proportion was Beijing genotypes followed by the orphan genotypes, T clade (which has three subclades each), MANU2, in which the proportion with H3 clade was approximately similar with U clade. The mean age (± S.D.) for these patients was approximately 41 years (41 ± 16), and 164 (60.7%) were male. These characteristics did not differ significantly between the sexes. A recent survey showed that the average age of patients with MDR-TB was 39.1 ± 16 years; our results are similar to those reported previously [13]. Furthermore, after stratification of the analysis by sex or age, there was still no association between Beijing strains and drug resistance (Table 3).

**Table 2:** Characteristics of patients infected with Beijing and non-Beijing isolates of *Mtb*

Characteristic	Number (%) of total isolates (*n*=270)	Number (%) of subgroups of isolates		OR (95% CI)	*P*-value
		Beijing, *n*=199	Non-Beijing, *n*=71		
**Drug resistance profile**					
Drug-resistance	102 (37.8%)	76 (38.2%)	26 (36.6%)	1.069 (0.610–1.874)	0.815
SM	39 (14.4%)	30 (15.1%)	9 (12.7%)	1.223 (0.550–2.721)	0.621
INH	75 (27.8%)	57 (28.6%)	18 (25.4%)	1.182 (0.638–2.190)	0.595
RIF	56 (20.7%)	44 (22.1%)	12 (16.9%)	1.396 (0.689–2.825)	0.353
EMB	34 (12.6%)	27 (13.6%)	7 (9.9%)	1.435 (0.596–3.458)	0.419
PZA	50 (18.5%)	40 (20.1%)	10 (14.1%)	1.535 (0.723–3.259)	0.263
MDR (INH+RIF)	52 (19.3%)	41 (20.6%)	11 (15.5%)	1.597 (0.650–3.926)	0.305
**Sex**					
Men	164 (60.7%)	116 (58.3%)	48 (67.6%)	1.493 (0.843–2.644)	-
Female	106 (39.3%)	83 (41.7%)	23 (32.4%)		0.168
**Age group**					
>30	187 (69.3%)	135 (67.8%)	52 (73.2%)	0.771 (0.421–1.410)	-
<30	83 (30.7%)	64 (32.2%)	19 (26.8%)		0.397

Abbreviations: CI, confidence interval; OR, odds ratio.

**Table 3:** Associations between Beijing strains and drug resistance, stratified by age or sex

Drug resistance	Age	Total cases, (number)	Beijing genotype, (number)	OR (95% CI)	Sex	Total Cases, (number)	Beijing genotype, (number)	OR (95% CI)
SM	>30	21	14	0.776 (0.294–2.047)	Male	24	18	1.286 (0.477–3.468)
	<30	18	16	2.566 (0.535–12.312)	Female	14	12	1.775 (0.368–8.565)
INH	>30	52	37	0.931 (0.458–1.893)	Male	49	36	0.989 (0.471–2.078)
	<30	23	20	2.424 (0.634–9.273)	Female	26	21	1.219 (0.403–3.691)
RIF	>30	38	28	1.099 (0.491–2.459)	Male	38	28	1.209 (0.535–2.735)
	<30	18	16	2.833 (0.589–13.627)	Female	18	16	2.507 (0.532–11.808)
EMB	>30	22	17	1.354 (0.473–3.881)	Male	21	16	1.376 (0.474–3.995)
	<30	12	10	1.574 (0.314–7.899)	Female	13	11	1.604 (0.329–7.812)
PZA	>30	35	25	0.955 (0.423–2.156)	Male	33	25	1.374 (0.571–3.307)
	<30	15	15	1.388 (1.197–3.259)	Female	17	15	2.316 (0.489–10961)
MDR (INH+RIF)	>30	46	36	1.527 (0.694–3.359)	Male	35	26	1.252 (0.537–2.917)
	<30	17	16	6.000 (0.741–48.59)	Female	17	15	2.316 (0.489–10.961)

Abbreviations: CI, confidence interval; OR, odds ratio.

### Stratified analyses

Statistical analyses were performed with the SPSS 20.0 statistical package. The Pearson chi-square test was used to compare the proportions of Beijing and non-Beijing genotypes in *Mtb* isolates with different drug susceptibility characteristics and determine statistical significance.

## Discussion

Although our study used a relatively small sample size, this is the research to investigate patient structure and its association with genotype and DST profiles in Yunnan Province. A study from Chen et al. [14] probed the molecular epidemiological characteristics of Mtb strains collected in Yunnan Province in 2014, and found that Beijing genotype is the main genotype of Yunnan Province, which is similar to our results. Another study from Li et al. [15] identified 24 types of mutations (including six novel mutations) after screening hotspot mutation regions of six first-line candidate drug resistance genes of 523 Mtb isolates collected in Yunnan Province in 2013–2015. Our current research integrated molecular genotyping into DR screening to analyze 270 Mtb strains in 2014–2016, and found that of 270 Mtb strains, 38 isolates belong to unnamed new genotypes, of which 17 isolates are drug-resistance isolates, which provide more wider and deeper integrative insights into the molecular epidemiological characteristics and DR status of Mtb strains in Yunnan. Similar to the results from different local regions in China, to some extent, our findings confirmed that the increased mutation rate of *Mtb* over time in this region, and Beijing family isolates still constitute the predominant MTB genotype in Yunnan [16]. Recent studies based on population-based molecular epidemiologic studies in China reported two main findings: (i) Beijing strains had higher odds of being in a genotypic cluster reflecting recent transmission but were not more likely than non-Beijing strains to be associated with drug resistance [17]; and (ii) Beijing genotypes were not more susceptible than non-Beijing isolates to show drug resistance from various geographic areas [17]. Beijing family epidemic genotypes show wide worldwide distribution, and as the major epidemic genotype, the frequency of its DR strains is significantly higher than that of non-Beijing genotypes; however, there is no direct evidence to indicate the relationship between Beijing genotype and drug resistance. One possible explanation for this situation might be that it is advantageous to the Beijing strains to spread. Yang et al. [17] also showed that no correlation has been found between drug resistance and sublineages. Concurrently, each social factor has different impacts on TB prevalence (especially MDR-TB) depending on the different geographical settings in China and after considering other factors such as sampling schemes and biases.

Researchers have demonstrated that the Beijing lineage is the predominant lineage in China, followed by the Euro-American lineage [18]. Our study also showed that Beijing strains are the dominant genotype and particularly are the dominant DR genotype in Yunnan. MANU2 (formerly only distributed in India) was first discovered in India, but T and U are widely distributed in Africa, Europe, and Central and South America as the essential epidemic strains. Among these strains, T1 and T2 are mainly epidemic in Africa and Central and South America [19]. H3 family is rarely distributed in Asia, and 60% of the H3 family is distributed in Armenia, Austria, and other countries. These non-Beijing strains are rarely distributed in Asia; however, our study found that H3 (0.37%), MANU (3.70%), T (7.41%), and U (0.74%) might reflect the potential recent ongoing transmission in China. Importantly, 42% non-Beijing strains (26 strains) were DR, which included 17 orphan genotype strains. Remarkably, eight orphan genotype strains were MDR, indicating that one out of two orphan genotype strains consisted of MDR. This finding also indicated that the new genotypes have an epidemic risk for MDR. Economic globalization, convenient travel, and tourism have all increased the possibility of gene mutation.

Several studies have shown that among all patients with TB, approximately one out of four had disease that was resistant to INH, RIF, or both, and one out of ten had MDR-TB [1]. Yunnan is one of the DR-TB hotspots in China, thus the increase in drug-resistance MTB rates remains a serious problem in Yunnan. In our study, clinical isolates were approximately one in four who had disease that was resistant to INH (75 [27.8%]). Our results are consistent with other studies. After applying either univariate or multivariate analysis, Yang et al. [17] observed that Beijing strains were significantly associated with INH or RIF resistance or were MDR.

We should expand the sample size and use a higher number of MDR isolates in our next study, research the relationship between genotypes and drug-resistance more thoroughly and accurately, and use more discriminating measurement methods such as mycobacterial interspersed repetitive unit–variable number tandem repeat analysis and whole genome sequencing that would enable typing to the strain level. Nevertheless, our study should help with the construction of a preventive program for controlling MDR-TB spread.

## Abbreviations

DNA: deoxyribonucleic acid
DR: drug-resistant
DR-TB: DR tuberculosis
DST: drug susceptibility testing
EMB: ethambutol
INH: isoniazid
MDR: multidrug-resistant
MDR-TB: MDR-tuberculosis
Mtb: *Mycobacterium tuberculosis*
PZA: pyrazinamide
RIF: rifampin
SM: streptomycin
TB: tuberculosis
TE: Tris/EDTA
